# The prevalence of Carbapenem Resistance Gram negative pathogens in a Tertiary Teaching Hospital in Jordan

**DOI:** 10.1186/s12879-023-08610-4

**Published:** 2023-09-27

**Authors:** Khawla Abu Hammour, Rana Abu-Farha, Rania Itani, Samar Karout, Aya Allan, Qusai Manaseer, Walid Abu Hammour

**Affiliations:** 1 Department of Clinical Pharmacy and Biopharmaceutics, Faculty of pharmacy, University of Jordan, Amman, Jordan; 2 Department of Clinical Pharmacy and Therapeutics, Faculty of Pharmacy, Applied Science Private University, 11931 Amman, Jordan; 3 Pharmacy Practice Department, Faculty of Pharmacy, Beirut Arab University, Riad El Solh, 1107 2809, 11-5020 Beirut, Lebanon; 4 Pharmacy Department, Jordan University Teaching Hospital, Amman, Jordan; 5 Department of Orthopedic surgery, Jordan University Teaching Hospital, Amman, Jordan; 6 Al Jalila Children’s Hospital, Dubai, United Arab Emirates

**Keywords:** Susceptibility Patterns, Multidrug Resistance, Antibiotics, Carbapenem-resistance, Gram-negative bacteria, Jordan

## Abstract

**Background:**

With the absence of new antimicrobial drugs being developed to replace those facing resistance, bacterial resistance continues to grow. Despite previous studies conducted in various countries, there is a lack of comprehensive local reporting on the occurrence of carbapenem resistance among gram-negative bacteria.

**Objective:**

This study aims to identify the prevalence of carbapenem-resistant gram-negative bacterial isolates.

**Method:**

A retrospective cross-sectional study was conducted at an academic hospital in Jordan over an eight-month period, spanning from November 2021 to June 2022. The study involved screening electronic medical records to identify patients with clinical cultures showing the growth of Gram-negative bacteria. Antimicrobial susceptibility results of the Gram-negative isolates were recorded.

**Results:**

A total of 1,043 isolated Gram-negative bacteria were analyzed for carbapenem susceptibility. Among the species tested, the most common carbapenem-resistant bacteria were *Acinetobacter baumannii* (153/164, 93.3%), followed by *Klebsiella pneumonia* (184/311, 59.2%), and *Pseudomonas aeruginosa* (67/160, 41.9%). The least commonly isolated species resistant to carbapenem were *Escherichia coli* (25/361, 6.9%) and *Proteus mirabilis* (1/30, 3.3%). None of *Serratia marcescens* or *Proteus vulgaris* isolates were resistant to carbapenem (0%). Overall, the prevalence of carbapenem-resistance gram-negative isolates was 41.2% (430 out of 1,043).

**Conclusion:**

This study provides population-specific data that are crucial for guiding empirical antimicrobial treatment decisions not only within the participating hospital but also in other nearby healthcare facilities. The results underscore the urgent need for coordinated efforts to address antibiotic resistance in Jordan. Comprehensive measures such as strict infection control methods, annual nationwide surveillance programs, and effective antimicrobial stewardship programs at the national level are imperative to reduce the overuse of broad-spectrum antibiotics.

## Background

Over the past 70 years, the successive discoveries of numerous antibiotics and the subsequent increase in antibiotic resistance have set the antimicrobial era apart. Antimicrobial resistance (AMR), which is linked to higher rates of morbidity and mortality, is largely influenced by the abuse of antibiotics [1–3]. AMR is particularly alarming in low- to middle-income countries, like Jordan, because of the recent growth in inappropriate use of licensed antimicrobial medicines [4–7]. According to research by Abu Hammour and colleagues, several multidrug-resistant organisms were discovered in tissue samples from 33 out of 127 individuals treated for community-acquired pneumonia or hospital-acquired illnesses. A significant prevalence of overall resistance of 26.0% resulted as a result compared to the typical resistance frequency of European countries participating in the Global PPS in 2015 [8].

Bacterial resistance is still spreading, and neither pharmaceutical corporations nor academic research facilities are producing new drugs to take the place of the antimicrobials against which resistance already exists. Determining the effect on actual infection rates is difficult [9, 10]. Globally coordinated actions are required in response to recent changes in the development of antibiotic resistance because of the potential health implications they could pose in several health sectors. Alarming global data demonstrate the prevalence of diseases brought on by common and diverse pathogenic microorganisms that have evolved resistance to antibiotics..

Carbapenems are effective against a wide range of bacteria. The unique structure of carbapenems, which provides protection against the majority of lactamases, including metallo- and extended spectrum -lactamases, is due to the interaction between the carbapenem and a -lactam ring [11]. In light of the fact that carbapenems are among the most successful drugs for treating infections caused by bacteria, the emergence and spread of antibiotic resistance to these drugs is a serious public health issue [12–14].

Although previous research has looked into the carbapenem resistance patterns of isolates in various different nations around the world, there haven’t been any in-depth local reporting on the incidence of carbapenem resistance in gram-negative bacteria. The objective of the current study was to determine the prevalence of carbapenem-resistant gram-negative bacteria isolated from clinical cultures in patients at the tertiary referral Jordanian hospital..

## Methods

### Study design and setting

The current study was conducted at Jordan University Hospital (JUH), the country’s first teaching referral tertiary healthcare facility. Jordanian population and their families can receive medical care at JUH. Data on patient demographics and antibiotic susceptibility tests were extracted from the microbiology lab records of hospitalized patients over an eight-month period, from the start of November 2021 to the end of June 2022, for a retrospective cross-sectional analysis. Patients whose clinical cultures for gram negative bacteria were positive make up the study’s final cohort. In vitro carbapenem susceptibility results for the different gram-negative isolates using the detection method based on the Vitec 2 System using agar dilution to determine the MICs were included in the analysis. This method is widely recognized and has been previously described in the literature [15]. A microorganism is considered resistant if it displays resistance to any of the carbapenem drugs used, including imipenem, meropenem, or ertapenem [16].

### Ethics approval and consent to participate

The study was designed and conducted in accordance with the guidelines outlined in the World Medical Association Declaration of Helsinki. The Institutional Review Board (IRB) of Jordan University Hospital (JUH) granted approval for the study’s protocol. The conduct of this investigation adhered to JUH’s code of ethics for protecting patients’ rights in research studies (number 10/2022/17082). To safeguard patients’ privacy and confidentiality, their medical records were anonymized and de-identified. The research team maintained no direct contact or follow-up with the patients. Given the retrospective observational nature of the study, where data collection occurred after patients’ discharge or death, and no patient identifiers were accessed, the IRB at JUH waived the need for informed consent.

### Data analysis

Data were analyzed using the 24th version of the Statistical Package for the Social Science (SPSS®, IBM Corp., Armonk, NY, USA). Frequencies and percentages were used to express categorical variables.

## Results

During the study period, from the 1st of November 2021 until the end of June 2022, 602 patients infected with gram negative bacteria were identified. Among them, 54.8% (n = 330) were females. Screening of the various clinical specimens for the included patients revealed 1,043 gram negative pathogens isolated over the eight-months study period as shown in Table 1.

**Table 1:** Number of gram negative pathogens isolated from various specimens at different months during the study period (n = 1,043)

Month	Number of gram-negative pathogens identified (%)
November 2021	103 (9.9)
December 2021	150 (14.4)
January 2022	121 (11.6)
February 2022	142 (13.6)
March 2022	143 (13.7)
April 2022	128 (9.0)
May 2022	107 (10.3)
June 2022	149 (14.3)
Total number of pathogens	1,043 (100)

Around 40% of the pathogens were isolated from urine samples (n = 413, 39.6%), followed by blood samples (n = 137, 13.1%), tracheal trap swab (n = 133, 12.8%), sputum (n = 121, 11.6%), and skin swab (n = 69, 6.6%) whereas clinical samples of peritoneal fluid, nasal swab and bile exhibited less prevalence of gram negative pathogens (≤ 1.2%) (Table 2**)**.

**Table 2:** A profile of clinical samples used as a source of the gram negative pathogens (n = 1,043)

Name of specimen	Number of pathogens
Urine Blood Bronchoalveolar lavage Catheter tip Nasal swap Peritoneal fluids Skin swap Sputum Tissue Tracheal trap swab Wound Bile Eye swap	413 (39.6) 137 (13.1) 20 (1.9) 20 (1.9) 4 (0.4) 12 (1.2) 69 (6.6) 121 (11.6) 38 (3.6) 133 (12.8) 55 (5.3) 9 (0.9) 12 (1.2)

**Fig. 1:**
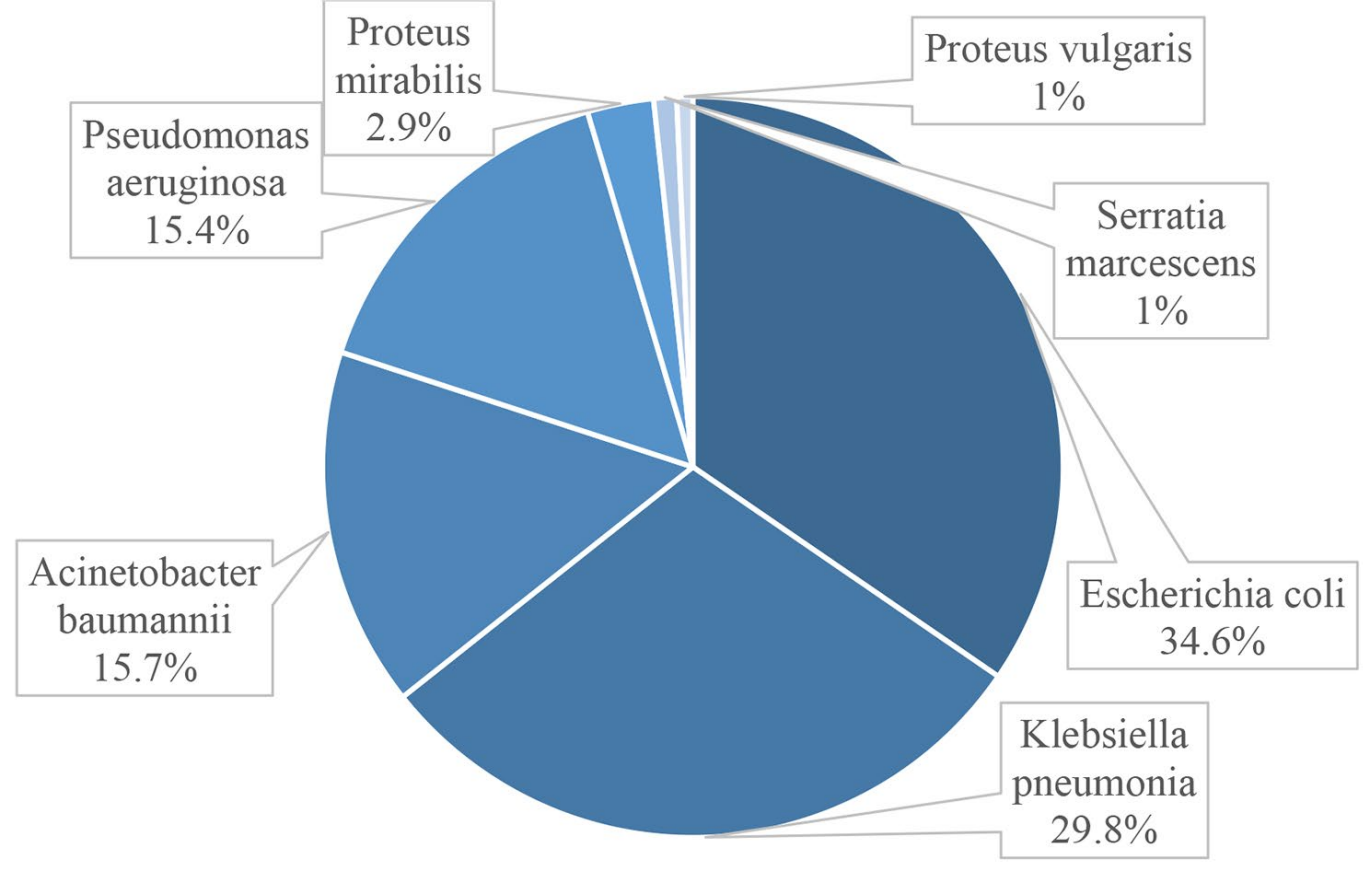
Proportion of Gram-negative isolates (n = 1,043)

The most common Gram-negative species isolated **(**Fig. 1) were *Escherichia coli* (n = 361, 34.6%), followed by *Klebsiella pneumonia* (n = 311, 29.8%), *Acinetobacter baumannii* (n = 164, 15.7%), and *Pseudomonas aeruginosa* (n = 160, 15.4).

The carbepenem susceptibility results of 1,043 isolated Gram-negative bacteria were analyzed. The isolates are summarized in Tables 3 and 4. The most common species resistant to carbapenem were *Acinetobacter baumannii* (153/164, 93.3%), followed by *Klebsiella pneumonia* (184/311, 59.2%), and *Pseudomonas aeruginosa* (67/160, 41.9%). The least isolated species that were resistant to carbapenem were *Escherichia coli* (25/361, 6.9%) and *Proteus mirabilis* (1/30, 3.3%). None of *Serratia marcescens* or *Proteus vulgaris* isolates were resistant to carbapenem (0%).

The overall prevalence of carbapenem resistance gram-negative isolates was 41.2% (430/1,043). Of these carbapenem resistant isolates (430), 42.7% were *Klebsiella pneumonia* (184/430), 35.5% were *Acinetobacter baumannii* (153/430), 15.5% were *Pseudomonas aeruginosa* (67/430), and 5.8% were *Escherichia coli* (25/430).

**Table 3:** The carbapenem susceptibility results of a total 1,043 gram-negative bacteria

Gram-negative isolates	Total number collected	R	S	UNS	R%^a^	R%^b^
*Escherichia coli*	361	25	227	109	6.9	5.8
*Klebsiella pneumonia*	311	184	76	51	59.2	42.7
*Acinetobacter baumannii*	164	153	9	2	93.3	35.5
*Pseudomonas aeruginosa*	160	67	51	42	41.9	15.6
*Proteus mirabilis*	30	1	20	9	3.3	0.2
*Serratia marcescens*	10	0	10	0	0	0
*Proteus vulgaris*	7	0	6	1	0	0
**Total Gram-negative isolates**	**1,043**	**430**	**399**	**214**	**-**	**41.2**

a: resistance rate within the same class of gram-negative isolate (R/total number collected), b: the prevalence of resistance among carbapenem resistant isolates (R/Total number of resistant gram-negative isolates (430)

**Table 4:** In vitro carbapenem susceptibility of the gram negative isolates (n = 1,043)

Gram-negative isolates	Number of isolates	Imipenem		Meropenem		Ertapenem	
		Resistant n (%)	Sensitive/Unknown n (%)	Resistant n (%)	Sensitive/Unknown n (%)	Resistant n (%)	Sensitive/Unknown n (%)
***Escherichia coli***	361	17 (4.7)	344 (95.3)	12 (3.3)	349 (96.7)	12 (3.3)	349 (96.7)
***Klebsiella pneumonia***	311	75 (24.1)	236 (75.9)	82 (26.4)	229 (73.6)	74 (23.8)	237 (76.2)
***Acinetobacter baumannii***	164	131 (79.9)	33 (20.1)	137 (83.5)	27 (16.5)	122 (74.7)	42 (25.6)
***Pseudomonas aeruginosa***	160	34 (21.2)	126 (78.8)	39 (24.4)	121 (75.6)	34 (21.2)	126 (78.8)
***Proteus mirabilis***	30	1 (3.3)	29 (96.7)	1 (3.3)	29 (96.7)	0 (31.0)	30 (100.0)
***Serratia marcescens***	10	0 (0.0)	10 (100.0)	0 (0.0)	10 (100.0)	0 (0.0)	10 (100.0)
***Proteus vulgaris***	7	0 (0.0)	7 (100.0)	0 (0.0)	7 (100.0)	0 (0.0)	7 (100.0)

## Discussion

Antimicrobial resistance has emerged as one of the most pressing global health threats of the 21st century, jeopardizing the hard-earned advancements in modern medicine. Among the numerous challenges posed by AMR, the rise in carbapenem resistance stands out as a serious concern due to the limited availability of effective and safe alternative antimicrobial treatments. Recognizing the rapid emergence and dissemination of carbapenem-resistant gram-negative pathogens is a significant clinical challenge. The potency of carbapenems against multidrug-resistant gram-negative pathogens has long been established, making the rise in resistance even more alarming [17–19]. Moreover, the dearth of viable alternative antibiotics exacerbates the difficulty in containing multidrug-resistant strains, thereby endangering the progress made in treating critically ill hospitalized patients, where the risk of acquiring multidrug-resistant infections is particularly high. Consequently, it is imperative to gain a comprehensive understanding of the prevalence of carbapenem resistance among gram-negative bacteria to formulate effective control strategies and curtail the spread of AMRs [17]. In light of this urgent need, the present study described the carbapenem susceptibility patterns among Gram-negative bacterial isolates in a tertiary care hospital, providing crucial insights into the current landscape of carbapenem resistance and paving the way for proactive interventions.

In Jordan, combating AMR has been an ongoing endeavor, but uncovering the true extent of the problem has been hindered by a scarcity of accessible epidemiological data. The absence of robust national surveillance programs, which would regularly report the incidence of multidrug-resistant organisms, compounds the challenge of identifying the local carbapenem resistance among Gram-negative isolates. This study has triumphed in shedding light on this issue, revealing an alarmingly high prevalence rate of 41% for carbapenem resistance among Gram-negative pathogens. Our findings were in line with a recent multicenter retrospective study conducted in Saudi Arabia between 2016 and 2020 [20]. The overall carbapenem resistance rate of the Gram-negative bacteria was 38% for imipenem and 46% for meropenem [20]. This situation is quite concerning and may be attributed to the increased utilization of carbapenems and the inadequate implementation of antibiotic stewardship. For instance, a nationwide multicenter Chinese study demonstrated the correlation between the prevalence of carbapenem-resistant Gram-negative bacteria and the intensity of antibiotic usage. Specifically, the study found that an increased utilization of meropenem was associated with higher rate of carbapenemase production [21].

*Acinetobacter baumannii, Klebsiella pneumonia*, and *Pseudomonas aeruginosa* topped the list of carbapenem-resistant species. Similarly, a surveillance study conducted in the United States screened infections caused by the four Gram-negative species between 2009 and 2013, namely *Acinetobacter baumannii, Pseudomonas aeruginosa, Klebsiella pneumoniae, and Escherichia coli. *This study revealed the prevalence rate of 4.5% for carbapenem resistance (13,262 out of 292,742 infections). Among these carbapenem-resistant infections, 60.3% were caused by *Pseudomonas aeruginosa*, 22% by *Acinetobacter baumannii*, and 17.7% were caused by *Klebsiella pneumonia* [22]. Furthermore, a recent multicenter study in Saudi Arabia illustrated the highest level of carbapenem resistance among *Acinetobacter baumannii* (92%) and *Pseudomonas aeruginosa* (88%) isolates. While *Klebsiella pneumoniae* (37%), and *Escherichia coli* (14%) exhibited a lower level of resistance [20]. Indeed, the World Health Organization (WHO) has issued a global priority list of antibiotic-resistant bacteria that require critical attention for the research and development of new antibiotics. Three of the four designated pathogens on this list include carbapenem-resistant Enterobacteriaceae (CRE), carbapenem-resistant *Pseudomonas aeruginosa*, and carbapenem-resistant *Acinetobacter baumannii* [23]. The threat posed by the carbapenem-resistant gram negative pathogens is due to their increasing incidence, the absence of safe and effective alternative antibiotics, and their high association with morbidity and mortality [23]. Therefore, the high carbapenem resistance rate among *Acinetobacter baumannii*, *Pseudomonas aeruginosa*, and *Klebsiella pneumoniae* should serve as a guide for healthcare providers discouraging the use of carbapenems as empirical antimicrobial therapy for infections caused by these pathogens, especially in the case of life-threatening infections.

Our study findings revealed intriguing patterns of carbapenem resistance among a range of clinically relevant bacterial strains, including *E. coli*, *K. pneumoniae*, *A. baumannii*, and *P. aeruginosa*. Notably, among these strains, ertapenem resistance exhibited lower levels compared to imipenem resistance across *E. coli* isolates. This observation raises important questions about the complex interplay of factors that influence carbapenem resistance in these isolates. It is evident that a multitude of variables, including genetic determinants and regulatory mechanisms, influence these resistance profiles [17]. To elucidate the underlying molecular pathways responsible for this observed difference, further in-depth research is warranted. We propose that exploring the roles of specific genes and enzymes, particularly those associated with carbapenemase production or efflux pump activity, holds promise for shedding light on the diverse resistance patterns exhibited by these bacterial strains. Investigating the genetic and phenotypic variations in these key elements may unveil novel insights into the mechanisms underpinning these resistance disparities.

### Limitations of the study

This study is subject to several limitations. Firstly, in our study, we primarily focused on the data extracted from the microbiology lab records of hospitalized patients whose clinical cultures for gram-negative bacteria were positive. This data mainly included information such as patients’ gender, date of hospitalization, type of tested specimen, and the results of antimicrobial susceptibility testing. While these data elements were crucial for our analysis of antibiotic susceptibility patterns, we acknowledge that our study did not include the extraction of specific patient clinical data. The absence of specific patient clinical data may have hindered our ability to identify and analyze risk factors associated with carbapenem-resistant Gram-negative bacteria. Clinical data such as comorbidities, prior antibiotic exposure, invasive procedures, and other relevant variables could provide valuable insights into the factors contributing to resistance. Secondly, the study was conducted in a single hospital in Jordan with a relatively small sample size, which limits the generalizability of the findings to other Jordanian hospitals. Therefore, future large-scale national multicenter studies should be conducted among patients infected with Gram-negative bacteria to ascertain the local prevalence of carbapenem resistance and identify associated risk factors. Such studies will offer valuable guidance to health authorities and policymakers in developing effective interventions to control the spread of carbapenem resistance and mitigate the associated morbidity and mortality.

## Conclusions

This study generated population-specific data that are crucial for guiding the selection of empirical antimicrobial treatment, not only within the participating hospital but also in other local healthcare facilities. These findings highlight the pressing need for collaborative efforts to address antimicrobial resistance in Jordan. They emphasize the urgent requirement to implement comprehensive measures such as strict infection control protocols, annual nationwide surveillance programs, and effective antimicrobial stewardship programs at the national level, aiming to limit the unnecessary use of broad-spectrum antibiotics.

## Data Availability

Not applicable.

## Data Availability

The dataset presented in this article is available only upon reasonable request since it contains confidential information. Requests to access the datasets should be directed to the corresponding author RI (r.itani@bau.edu.lb).
